# Comparison of the amine/amino acid activation profiles of the β- and γ-carbonic anhydrases from the pathogenic bacterium *Burkholderia pseudomallei*

**DOI:** 10.1080/14756366.2017.1387544

**Published:** 2017-11-03

**Authors:** Daniela Vullo, Sonia Del Prete, Sameh M. Osman, Fatmah A. S. Alasmary, Zeid AlOthman, William A. Donald, Clemente Capasso, Claudiu T. Supuran

**Affiliations:** a Dipartimento Di Chimica, Laboratorio di Chimica Bioinorganica, Polo Scientifico, Università degliStudi di Firenze, Florence, Italy;; b CNR, Istituto di Bioscienze e Biorisorse, Napoli, Italy;; c Dipartimento Neurofarba, Sezione di Scienze Farmaceutiche e Nutraceutiche, Università degli Studi di Firenze, Florence, Italy;; d Department of Chemistry, College of Science, King Saud University, Riyadh, Saudi Arabia;; e School of Chemistry, University of New South Wales, Sydney, New South Wales, Australia

**Keywords:** Carbonic anhydrase, metalloenzymes, pathogens, activators, *Burkholderia pseudomallei*

## Abstract

The β-class carbonic anhydrase (CA, EC 4.2.1.1) from the pathogenic bacterium *Burkholderia pseudomallei*, BpsCAβ, that is responsible for the tropical disease melioidosis was investigated for its activation with natural and non-natural amino acids and amines. Previously, the γ-CA from this bacterium has been investigated with the same library of 19 amines/amino acids, which show very potent activating effects on both enzymes. The most effective BpsCAβ activators were L- and D-DOPA, L- and D-Trp, L-Tyr, 4-amino-L-Phe, histamine, dopamine, serotonin, 2-pyridyl-methylamine, 1-(2-aminoethyl)-piperazine and L-adrenaline with K_A_s of 0.9–27 nM. Less effective activators were D-His, L- and D-Phe, D-Tyr, 2-(2-aminoethyl)pyridine and 4-(2-aminoethyl)-morpholine with K_A_s of 73 nM–3.42 µM. The activation of CAs from bacteria, such as BpsCAγ/β, has not been considered previously for possible biomedical applications. It would be of interest to perform studies in which bacteria are cultivated in the presence of CA activators, which may contribute to understanding processes connected with the virulence and colonization of the host by pathogenic bacteria.

## Introduction

Carbonic anhydrases (CAs, EC 4.2.1.1) are a superfamily of ubiquitous metalloenzymes with the catalytically active form represented by a metal hydroxide derivative acting as a potent nucleophile^1–15^. They catalyze a simple but physiologically relevant reaction in which carbon dioxide is reversibly hydrated to bicarbonate and protons^2^^,^^5^^,^^6^^,^^13^^,^^15^. CAs are grouped in seven genetically distinct families, named α-, β-, γ-, δ-, ζ-, η- and ɵ-CAs, and share a relatively low similarity for both sequences and three-dimensional structures^5^^,^^6^^,^^8^^,^^13^^,^^15–17^. α-CAs are normally monomers and rarely dimers; β-CAs are dimers, tetramers or octamers; γ-CAs are trimers^18–21^. Interesting, ζ-CA has three slightly different active sites on the same polypeptide chain^5^^,^^8^^,^^15^^,^^16^^,^^22–25^. X-ray crystal structures of δ-, η- and θ-CAs are not available to date. The catalytic active sites of α-, β-, δ-, η- and, perhaps θ-CAs contain a Zn(II) ion. γ-CAs are Fe(II) enzymes but they are also active coordinating Zn(II) or Co(II) ions, whereas ζ-CAs bind Cd(II) or Zn(II) within their active site, being cambialistic enzymes^1^^,^^5^^,^^6^^,^^15^^,^^16^^,^^26^^,^^27^. The metal ion from the CA active site is coordinated by three His residues in the α-, γ-, δ- and probably the θ-classes: by one His and two Cys residues in β- and ζ-CAs or by two His and one Gln residues in the η-class, with the fourth ligand being a water molecule/hydroxide ion acting as nucleophile in the catalytic cycle of the enzyme^18^^,^^19^^,^^21^^,^^28–35^.

CAs are involved in many crucial physiologic and pathologic processes connected to pH regulation, secretion of electrolytes, biosynthetic processes, photosynthesis, tumorigenesis, etc. The existence in bacteria of genes encoding for CAs from at least one of the α-, β- and γ-classes suggests that these enzymes are essential for the bacterial physiology. In fact, it has been demonstrated that CAs are involved in the transport and supply of CO_2_ or HCO_3_^−^, pH regulation, acclimatization of the pathogen within the stomach, induction of the cholera toxin expression, pathogenicity and/or the growth of the microorganism, and, at least in *Escherichia coli,* in the cyanate degradation^16^^,^^36–44^. Generally, CAs hydrate carbon dioxide at a very high rate, with pseudo first order kinetic constants as high^15^^,^^16^ as 10^4^–10^6^ s ^–1^. The rate-determining step of the entire catalytic process for all CA genetic families is likely the formation of the metal hydroxide species of the enzyme, via the transfer of a proton from the metal-coordinated water molecule to the surrounding solvent to form the nucleophilic form of the enzyme^5^^,^^6^^,^^13^^,^^15^^,^^16^. As a consequence, in all CAs, a proton shuttle residue is present which controls the transfer of the proton from the active site to the protein surface^45^^,^^46^. For α-CAs, a His residue (His64, hCA I numbering system) plays the role of proton shuttle, whereas for other genetic families, the nature and the role of the proton shuttle are less well understood^25–31^. Thus, the manipulation of the proton shuttle within the CAs active sites is crucial to the function of these enzymes and explains the efficacy and prominence of the catalytic processes in which these enzymes participate^45^^,^^47^. A multitude of physiologically active compounds such as biogenic amines (histamine, serotonin, and catecholamines), amino acids, oligopeptides or small proteins were shown to act as efficient carbonic anhydrase activators (CAAs)^47^. CAAs may be useful in the treatment of Alzheimer’s disease, in aging, in achieving spatial learning and memory therapy^45^^,^^46^. Indeed, the action of CAAs can be mediated by extracellular signal-regulated kinase (ERK) pathways in a critical step for memory formation, within the cortex and the hippocampus, which are two brain areas involved in memory processing and rich in various CA isoforms^46^.

Whereas bacterial CA inhibitors (CAIs) were extensively studied, leading to a detailed understanding of the catalytic and inhibition mechanisms, only a few studies are available on the bacterial CAAs. Recently, our groups described the biochemical properties of a β- and γ-CA from the pathogenic bacterium *Burkholderia pseudomallei*, which is responsible for the tropical disease melioidosis^48–51^. These enzymes, called BpsCAβ and BpsCAγ, showed high catalytic activity for the physiologic CO_2_ hydration reaction to bicarbonate and protons (*k*_cat_ 10^5^ s^−1^)^48–51^. Moreover, the study of the inhibition profiles with the classical CA inhibitors (sulfonamides and anions) revealed an interesting structure–activity relationship for the interaction of these enzymes with the inhibitors^48–51^. We also investigated the activation profiles of BpsCAγ with a series of natural and non-natural amino acids and aromatic/heterocyclic amines^52^. Here, we report the effects the aforementioned CAAs on the activity of BpsCAβ, which has not yet been investigated for its activation profile. We also compare the effects of these classes of these CAAs on the β- and γ-class enzymes from this bacterium.

## Materials and methods

### Gene identification and cloning

The identification of the genes encoding *B. pseudomallei* β-CA (BpsCAβ) and γ-CA (BpsCAγ) was performed as described by Del Prete et al.^50^. Briefly, the β-CA gene with the accession number WP_004189176.1 and the γ-CA gene (accession number: WP_038762492.1) from *Burkholderia pseudomallei* were identified running the “BLAST” program, using the nucleotide sequences of known bacterial β-CAs or γ-CAs as query sequence. The GeneArt Company (Invitrogen, Carlsbad, CA), specializing in gene synthesis, designed the synthetic BpsCAβ (BpsCAβ-DNA) and BpsCAγ (BpsCAγ-DNA) genes encoding for the β- and γ-CAs and containing four base-pair sequences (CACC) necessary for directional cloning at the 5′ end of the BpsCAβ and BpsCAγ genes. The recovered BpsCAβ and BpsCAγ genes and the linearised expression vector (pET-100/D-TOPO) were ligated by T4 DNA ligase to form the expression vector pET-100/BpsCAβ or pET-100/BpsCAγ.

### Expression and purification

BL21-CodonPlus(DE3)-RIPL competent cells (Agilent, Palo Alto, CA) were transformed with pET-100/BpsCAβ or pET-100/BpsCAγ, grown at 37 °C, and induced with 1 mM IPTG. After 30 min, ZnSO_4_ (0.5 mM) was added to the culture medium and cells were grown for an additional 3 h. Subsequently, cells were harvested and re-suspended in the following buffer: 50 mM Tris/HCl, pH 8.0, 0.5 mM PMSF and 1 mM benzamidine. Cells were then disrupted by sonication at 4 °C. After centrifugation at 12,000× *g* for 45 min, the supernatant was incubated with His Select HF nickel affinity gel resin (Sigma, St. Louis, MO) equilibrated in lysis buffer for 30 min. Following centrifugation at 2000× *g*, the resin was washed in buffer (50 mM Tris/HCl, pH 8.3, 500 mM KCl, 20 mM imidazole). The protein was eluted with the wash buffer containing 300 mM imidazole. The collected fractions were dialyzed against 50 mM Tris/HCl, pH 8.3. At this stage of purification, the proteins were at least 95% pure and the obtained recovery was of about 2 mg of the recombinant proteins.

### Carbonic anhydrase activity assay and determination of the activation constant

An applied photophysics stopped-flow instrument was used for assaying the CA catalysed CO_2_ hydration activity^53^. Phenol red (at a concentration of 0.2 mM) was used as an indicator, working at the absorbance maximum of 557 nm, with 10 mM TRIS (pH 8.3) as buffer, 0.1 M Na_2_SO_4_ (for maintaining constant ionic strength), following the CA-catalysed CO_2_ hydration reaction for a period of 10 s at 25 °C. The CO_2_ concentrations ranged from 1.7 to 17 mM for the determination of the kinetic parameters and activation constants. For each activator, at least six traces of the initial 5–10% of the reaction have been used for determining the initial velocity. The unactivated rates were determined in the same manner and subtracted from the total observed rates. Stock solutions of activators **1–19** (10 mM) were prepared in distilled-deionized water and dilutions up to 0.01 nM were done thereafter with distilled-deionized water. Activator and enzyme solutions were preincubated together for 15 min at room temperature prior to assay, in order to allow for the formation of the E-A complex. The activation constant (*K*_A_), defined similarly with the inhibition constant K_I_^5–8^, can be obtained by considering the classical Michaelis–Menten equation (Equation (1)), which has been fitted by non-linear least squares by using PRISM 3:

(1) $$v=v_{\text{max}}/\{\text{1}+K_{\text{M}}/\left[\text{S}\right]\;\left(\text{1}+{\left[\text{A}\right]}_{\text{f}}/{\text{K}}_{\text{A}}\right)\}$$

where [A]_f_ is the free concentration of activator.

Working at substrate concentrations considerably lower than K_M_ ([S] ≪ K_M_), and considering that [A]_f_ can be represented in the form of the total concentration of the enzyme ([E]_t_) and activator ([A]_t_), the obtained competitive steady-state equation for determining the activation constant is given by Equation (2)^45^^,^^47^^,^^54^^,^^55^:

(2) $$v=v_{0}.{\text{K}}_{\text{A}}/\{{\text{K}}_{\text{A}}+({\left[\text{A}\right]}_{\text{t}}–0.\text{5}{\left\{({\left[\text{A}\right]}_{\text{t}}+{\left[\text{E}\right]}_{\text{t}}+{\text{K}}_{\text{A}})–{\left({\left[\text{A}\right]}_{\text{t}}+{\left[\text{E}\right]}_{\text{t}}+{\text{K}}_{\text{A}}\right)}^{\text{2}}–\text{4}{\left[\text{A}\right]}_{\text{t}}.\left[\text{E}\right]\text{t}\right)}^{\text{1}/\text{2}}\}\}$$

where *v*_0_ represents the initial velocity of the enzyme-catalyzed reaction in the absence of activator^45^^,^^47^^,^^54^^,^^55^.

## Results and discussion

**Figure 1.**
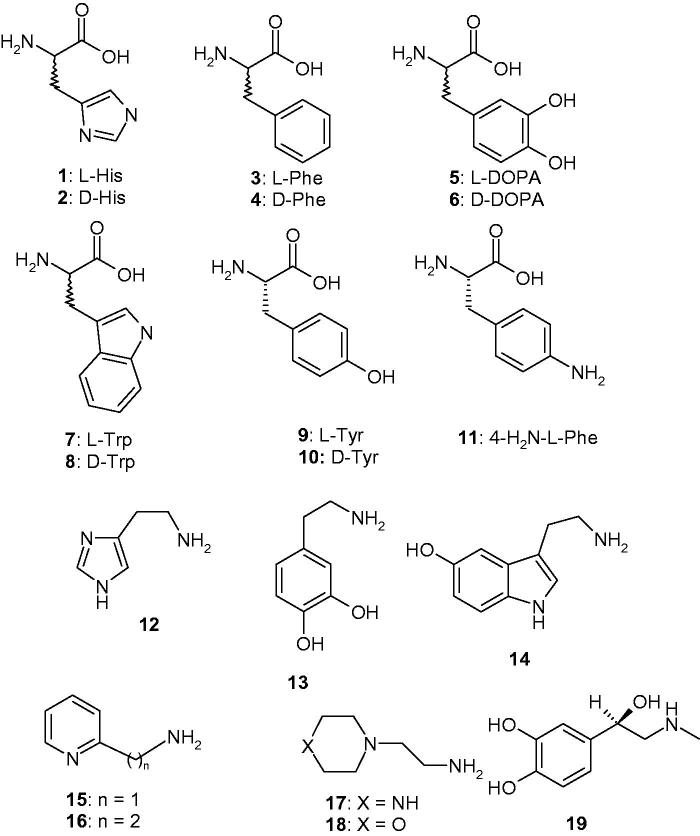
Chemical structures of compounds **1–19** investigated as CAAs in the present paper.

The activators **1**–**19** were included in this study (Figure 1), as they were employed for investigations as CAAs against many classes of CAs, including the bacterial BpsCAγ^52^. Both natural and non-natural amino acids and amines are among the investigated compounds (Figure 1)^53^.

L-Tyr (at 10 μM concentration) is a very effective CAA for all enzymes considered here, i.e. hCA I, II and BpsCAγ/β, that significantly enhances the *k*_cat_ values for each enzyme, whereas *K*_M_ remains unchanged (Table 1). This situation has been observed for all CAAs investigated so far^54–56^. L-Tyr is a nanomolar activator for the α-class enzymes (hCA I and II) and BpsCAβ with K_A_s in the range of 3–20 nM and a submicromolar activator for BpsγCA (Table 2). Owing to the very high efficacy as a BpsCAβ activator, L-Tyr increased the kinetic constant of this enzyme by a factor of 19.3 compared with the unactivated rate. This is one of the highest kinetic effects observed so far for an activator of these enzymes.

**Table 1.** Activation of human carbonic anhydrase (hCA) isozymes I, II and BpsCAγ/β with L-Tyr, at 25 °C, for the CO2 hydration reaction^53^.

	*k*_cat_^d^	*K*_M_^d^	(*k*_cat_)_L-Tyr_^e^	*K*_A_^f^ (μM)
Isozyme	(s^−1^)	(mM)	(s^−1^)	L-Tyr
hCA I^a^	2.0 × 10^5^	4.0	13.9 × 10^5^	0.020
hCA II^a^	1.4 × 10^6^	9.3	12.8 × 10^6^	0.011
BpsCAγ^b^	5.3 × 10^5^	21.2	13.8 × 10^5^	0.200
BpsCAβ^c^	1.6 × 10^5^	4.7	3.10 × 10^6^	0.003

a Human recombinant isozymes, from Ref.^7^.

b Bacterial recombinant enzyme, from Ref.^52^.

c Bacterial recombinant enzyme, this work.

d Observed catalytic rate without activator. *K*_M_ values in the presence and the absence of activators were the same for the various CAs (data not shown).

e Observed catalytic rate in the presence of 10 μM activator.

f The activation constant (*K*_A_) for each enzyme was obtained by fitting the observed catalytic enhancements as a function of the activator concentration^53^. Mean from at least three determinations by a stopped-flow, CO_2_ hydrase method. Standard errors were in the range of 5–10% of the reported values (data not shown).

**Table 2.** Activation constants of hCA I, hCA II and the bacterial CAs BpsCAγ/β with amino acids and amines **1–19**. Data for hCA I, II and BpsCAγ are from Refs.^25^^,^^52^.

			K_A_ (μM)^d^		
No.	Compound	hCA I^a^	hCA II^a^	BpsγCA^b^	BpsCAβ^c^
**1**	L-His	0.03	10.9	24.7	31.6
**2**	D-His	0.09	43	0.086	0.98
**3**	L-Phe	0.07	0.013	1.73	3.42
**4**	D-Phe	86	0.035	0.13	0.075
**5**	L-DOPA	3.1	11.4	0.072	0.009
**6**	D-DOPA	4.9	7.8	0.98	0.007
**7**	L-Trp	44	27	0.43	0.002
**8**	D-Trp	41	12	0.052	0.001
**9**	L-Tyr	0.02	0.011	0.20	0.003
**10**	D-Tyr	nt	nt	32.8	1.89
**11**	4-H_2_N-L-Phe	0.24	0.15	0.009	0.0009
**12**	Histamine	2.1	125	0.12	0.012
**13**	Dopamine	13.5	9.2	0.014	0.006
**14**	Serotonin	45	50	0.10	0.027
**15**	2-Pyridyl-methylamine	26	34	2.36	0.016
**16**	2-(2-Aminoethyl)pyridine	13	15	0.034	0.94
**17**	1-(2-Aminoethyl)-piperazine	7.4	2.3	0.018	0.004
**18**	4-(2-Aminoethyl)-morpholine 0.14	0.19	0.015	0.073	
**19**	L-Adrenaline	0.09	96	0.019	0.002

a Human recombinant isozymes, stopped flow CO_2_ hydrase assay method^25^.

b From Ref.^52^, stopped flow CO_2_ hydrase assay method.

c This work.

d Mean from three determinations by a stopped-flow, CO_2_ hydrase method^14^. Standard errors were in the range of 5–10% of the reported values (data not shown); nt: not tested.

In Table 2, the CAA profiles of the amino acids and amines **1–19** that were measured for two human (α-class) and two bacterial (γ-class) CAs (i.e. four diverse enzymes) are shown. The CAA profiles for hCA I, hCA II and BpsγCA were reported previously^25^^,^^52^. The amino acids/amines **1–19** are effective activators of BpsCAβ (Table 2). In fact, these amino acids and amines show activation constants ranging between 0.9 nM and 31.6 µM, leading to a very interesting structure-activity relationship, as outlined below:

- A large number of the investigated amino acids and amines showed extremely effective activating properties against BpsCAβ, with activation constants in the subnanomolar – low nanomolar range, more precisely of 0.9–27 nM. They include L- and D-DOPA, L- and D-Trp, L-Tyr, 4-amino-L-Phe, histamine, dopamine, serotonin, 2-pyridyl-methylamine, 1-(2-aminoethyl)-piperazine and L-adrenaline. 4-Amino-L-Phe **11** was the most effective, subnanomolar activator, followed by D-Trp, L-Trp, L-Tyr and L-adrenaline which showed *K*_A_s in the range of 1-3 nM. Although many of these derivatives also showed effective BpsCAγ activating properties,^52^ their effects on the β-class enzyme are more potent, as observed by comparing the *K*_A_s of these compounds against the two pathogenic enzymes. Furthermore, in many cases, these compounds were more effective in selectively activating the bacterial versus the human α-class CAs (Table 2). Again, the D-amino acid derivatives were more effective BpsCAβ activators compared with their L-enantiomer, except for Tyr, for which the L-enantiomer was a better activator compared with the D-enantiomer. Small changes in the scaffold (e.g. the presence of an amino moiety as in **11** or two OH groups as in DOPA) lead to an enhanced effect compared to the parent L-/D-Phe scaffold. In fact, D-Phe is a rather effective activator (*K*_A_ of 75 nM) whereas the L-enantiomer is a weak, micromolar activator with a *K*_A_ of 3.42 µM.
- Compounds with medium CAA potency for BpsCAβ were D-His, L- and D-Phe, D-Tyr, 2-(2-aminoethyl)pyridine and 4-(2-Aminoethyl)-morpholine, which have *K*_A_s ranging between 73 nM and 3.42 µM (Table 2). Again small differences in the scaffold of the activator lead to significant differences in activity. For example, the two amines **15** and **16** only differ by an extra CH_2_ moiety. However, **16** is 58.7 times less effective as a CAA compared with **15**. Compounds **17** and **18** are also structurally similar, with an oxygen atom in the ring of **17** being replaced by an NH group in **18**. However, the two compounds show a highly different action on BpsCAβ: the piperazine **17** was 18.2 times more effective as a CAA compared with the morpholine **18**.
- The least effective activator was L-His, with a *K*_A_ of 31.6 µM (Table 2). It may be observed that the D-enantiomer **2** was a much more effective activator (32.2 times) compared with the L-enantiomer **1**.

## Conclusions

The present study evidenced that many natural and non-natural amino acids and amines show very potent activating effects on both CAs present in this pathogenic bacterium. Among them are L- and D-DOPA, L- and D-Trp, L-Tyr, 4-amino-L-Phe, histamine, dopamine, serotonin, 2-pyridyl-methylamine, 1-(2-aminoethyl)-piperazine and L-adrenaline, which showed activation constants ranging between 0.9 and 27 nM. Less effective activators were D-His, L- and D-Phe, D-Tyr, 2-(2-aminoethyl)pyridine and 4-(2-aminoethyl)-morpholine, which showed *K*_A_s ranging between 73 nM and 3.42 µM. The activation of CAs in bacteria, such as BpsCAγ/β, has not been previously considered until now for possible biomedical applications. In fact, no growth studies of these bacteria in media which are enriched in CAAs have been reported in the literature. It would be of interest to perform such studies which may bring new light in understanding processes connected with the virulence and colonization of the host by such bacteria, which are responsible for a rather difficult to treat disease, melioidosis^56^. Moreover, biogenic CAAs may be useful as potential biomarkers for diagnosis of such pathogen born diseases to direct treatment and prevent sepsis.
